# Automated detection of polymicrogyria in pediatric patients using deep learning

**DOI:** 10.1038/s41598-025-25572-6

**Published:** 2025-11-24

**Authors:** Shagnik Guha, Venkatesh Bhandage, Aman Agarwal

**Affiliations:** Manipal Institute of Technology, Manipal Academy of Higher Education, Manipal, India

**Keywords:** Cortical structures, Deep learning, MRI images, Polymicrogyria, ResNet-50, ResNet-101, VGG-16, MobileNetV2, DenseNet-201, Medical imaging, Brain imaging, Neurological disorders, Paediatric neurological disorders, Engineering, Biomedical engineering

## Abstract

Polymicrogyria (PMG) is a multifaceted neurological disorder caused by abnormal cortical folding, mostly in children. It commonly results in developmental delays, seizures, and motor weakness. The mild features of PMG in neuroimaging often make its identification difficult, even for experts. In this paper, we assess the efficacy of various advanced image preprocessing strategies on the overall performance of Convolutional Neural Network (CNN) applied for PMG diagnosis in MRI brain scans. We employ a pre-processing sequence that includes Min–Max normalization, Contrast Limited Adaptive Histogram Equalization (CLAHE), Bilateral filtering, and Canny edge detection aimed at improving the recognition of subtle features without losing essential details. The techniques can enhance the visualization of delicate structural deformities in the brain MRI images and assist in the diagnosis of neurological disorders by clinicians. Experimental results suggest that performance enhancement was achieved with all of the tested CNN architectures. ResNet-101 has exhibited the most remarkable accuracy enhancement by 10.3%. ResNet and VGG architectures delivered much greater performance improvement as compared to MobileNetV2 and DenseNet-201 models. GradCAM++ is adopted to infer the decision-making mechanism of the considered deep learning architectures. The methodology finds applications in neurological imaging and may be used to assist healthcare providers in the diagnosis of polymicrogyria. Our findings emphasize the crucial role of image pre-processing techniques in increasing the capabilities of deep learning frameworks to assist with complex tasks in medical image analysis.

## Introduction

Neurological disorders are abnormal conditions that can affect the mental growth of human beings. Different neurological disorders include seizures, Parkinson’s disease, Alzheimer’s disease, Schizophrenia, and Epilepsy. Different neuroimaging techniques can be adapted for the study of brain disorders. Positron emission tomography (PET)^1^, magnetic resonance imaging (MRI)^2^, and computed tomography (CT)^3^ are the predominant techniques for the study of brain neurological disorders. Though neurological disorders can be detected by magnetic resonance imaging (MRI), there may be difficulties faced by radiologists in accurate detection of brain disorders. There is a need to leverage technological advancements to ease the process of abnormality detection and aid the treatment process. Different research has been carried out in the area of neurological disorder detection based on deep learning and machine learning techniques. Convolutional Neural Networks (CNNs) have been used for the analysis of brain MRI images for possible abnormality detection^4^.

**Fig. 1:**
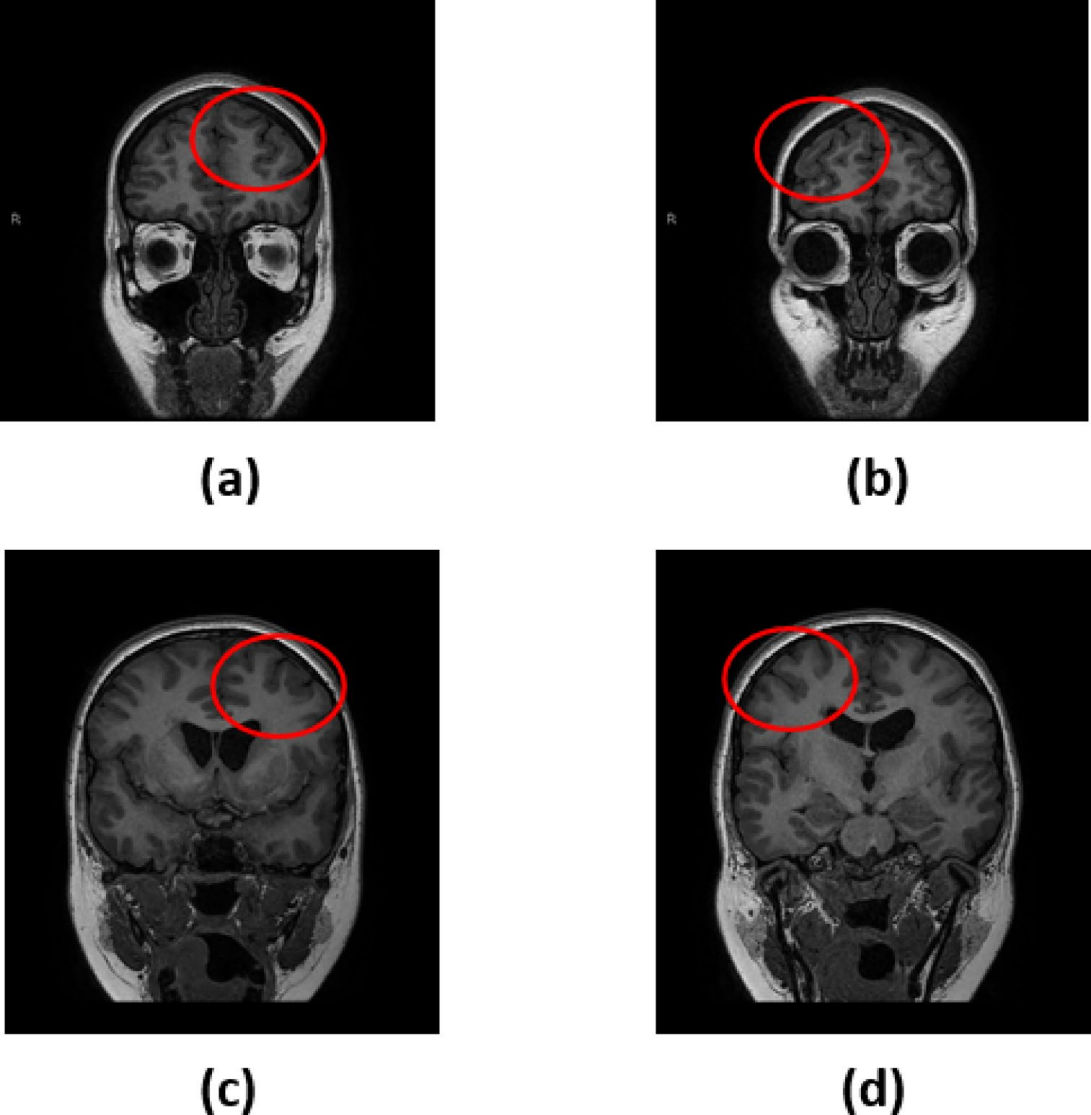
Images depicting the difference between polymicrogyria and healthy cases, (**a**)-(**b**) indicate the normal images with uniform and normal gray matter, highlighted in the red circle. (**c**)-(**d**) indicate the presence of polymicrogyria with irregular and thick gray matter.

Research on pediatric neurological disorders has been evolving due to the need to mitigate the growth of neurological disorders at an early stage of life. Different research is conducted in the area of pediatric neuroimaging^5^. Polymicrogyria (PMG) is a type of neurological disorder that is predominantly seen in the early stages of childhood. This can be related to motor weakness, developmental delays, and seizures. The characteristics of PMG include loss of gray-white matter differentiation, shallow sulci, the presence of many small gyri, and the existence of cerebral cortex with the presence of abnormal and thick gray matter^6^. The MRI images of PMG show the presence of irregular gray matter, and the MRI images of controlled subjects show the normal presence of gray matter. Figure 1 illustrates a few images of PMG and controlled cases. The variations in the presence of gray matter are shown in the ovals. The images are taken from the dataset openly made available by authors Jian Wang et al.^6^. In certain instances, distinguishing between the PMG image and a healthy image can be challenging, highlighting the need to create machine learning models to recognize PMG.

Tugba A. et al.^4^ have used a mix of 2D and 3D deep learning models to estimate the Myelin Maturation in brain images of children aged between 0 to 3 years. Jiang Wang et al.^5^ have provided a comprehensive review of different deep learning-based research conducted in pediatric neuroimaging. They have elaborated on brain maturation, brain development, abnormality detection, segmentation, classification, image processing, and image reconstruction. Major challenges being faced by all the researchers are the unavailability of standard datasets, data privacy, ethical concerns, and limited data availability. L. Zhang et al.^6^ have presented a deep learning (DL) based approach for the detection of Polymicrogyria (PMG), a cortical disorder often seen in children, using brain MRI images. They have created their dataset called the open pediatric polymicrogyria MRI (PPMR) dataset from the Children’s Hospital of Eastern Ontario (CHEO). They propose a center-based deep contrastive metric learning (cDCM) approach for detecting anomalies. They have achieved a precision of 71.86% and a recall of 88.07%.

Omneya Attallah et al.^7^ have employed machine learning based approaches to identify disorders in brain embryos. They have extracted the deep features from the images by using deep learning architectures, namely, ResNet50, AlexNet, and GoogleNet. The extracted features are fed to the Support Vector Machine (SVM) classifier for the classification of normal and abnormal brain images. They have also classified the images by combining the features obtained. A detailed review of the potential uses of machine learning models in the study of Epilepsy (EP) neurological disorder is given by D Sone and I. Beheshti^8^. Researchers have used Logistic Regression (LR), Random Forest (RF), SVM, Artificial Neural Networks (ANN), and Deep Learning (DL) models for the study of the Epilepsy brain disorder.

The usage of brain EEG signals for possible detection of neurological disorders is reported in research^9–13^, where EEG signals are pre-processed and classified into different brain abnormal conditions by using DL and ML techniques for conditions such as Parkinson’s Disease (PD), Epilepsy (EP), Schizophrenia (SZ), Alzheimer’s disease, and Autism Spectrum Disorder (ASD). A similar attempt is made by researchers Md. Nurul Ahad Tawhid et al.^14^ and^15^, where the EEG signals are converted into spectrogram images and classified using deep learning techniques, obtaining promising results with the spectrogram image-based classification approach. Md. Nurul Ahad Tawhid et al.^16^ extracted textural features from the EEG derived spectrogram images and classified them by using machine learning classifiers such as Support Vector Machine (SVM), k-nearest Neighbor (kNN), Linear Discriminant Analysis (LDA), and Random Forest (RF). Similarly, statistical features based on hypercube pattern and KNN classifier are utilized in the classification of Epileptic seizures^17^. While these EEG approaches operate in a different modality, they demonstrate that preprocessing can significantly enhance classification performance.

The summary of the most relevant works related to Polymicrogyria and other neurological disorders is given in Table 1. There is much scope to conduct research on the detection and classification of Polymicrogyria. The usage of different deep learning models for the effective classification of PMG can be explored. There is a scope to adopt pre-processing techniques on the PMG images for enhanced classification. We have attempted to apply various pre-processing techniques for achieving improved classification accuracy of PMG on this newly introduced dataset by L. Zhang et al.^6^.

**Table 1:** Gist of existing works related to polymicrogyria.

Reference	Datasets Used	Methodology adopted	Results Reported (%)
^6^	PPMR dataset	The CDCM loss function is used to classify PMG using ResNet50	Recall—88.07, Precision – 71.86
^7^	Embryonic brain dataset	Deep features are extracted and classified using SVM classifier	Accuracy – 87.7
^4^	Internal dataset, National Institute of Health (NIH) pediatric brain MRI database, and the Developing Human Connectome Project (dHCP) database	Combining 2D and 3D CNN into an ensemble to predict myelin maturation age. 3D CNN from^46^ and EfficientNet-b0 as the 2D CNN	Mean Absolute Error (MAE) Results: Cross-validation set: 2D model – 1.53, 3D model – 2.06, Ensemble model – 1.63 Internal test set: 2D model – 1.43, 3D model – 2.55, Ensemble model – 1.77 External NIH dataset: 2D model – 2.26, 3D model – 2.27, Ensemble model – 1.22 External dHCP dataset: 2D model – 0.44, 3D model – 0.27, Ensemble model – 0.31
^32^	Publicly available Brain Tumor MRI dataset	Transfer learning is adopted to evaluate and compare multiple pre-trained deep learning models, such as, VGG-16, Inception-v3, and ResNet50	Accuracies of VGG16 – 96, InceptionV3 – 78, ResNet50 – 95 Precision of VGG16 – 94, InceptionV3 – 75, ResNet50 – 92 Recall of VGG16 – 100, InceptionV3 – 70, ResNet50 – 89 F1-score of VGG16 – 98, InceptionV3 – 73, ResNet50 – 94
^34^	Custom MRI dataset collected and augmented by Swati Kanchan from NIT Durgapur	Image resizing and normalization techniques are adopted. Transfer learning and fine-tuning of MobileNet CNN are utilized. GradCam is experimented for visual explanation	Validation Accuracy: 97.24; Test Accuracy: 97.86; Precision: 97.91; Recall: 97.86; F1-score: 97.86 for four class classification

The objectives of the research are:

1. To explore the impact of image pre-processing techniques for detecting Polymicrogyria.
2. To develop deep learning models that can categorize brain MRI images into polymicrogyria and healthy cases.

Contributions to the research are:

1. Pre-processing methods such as min–max normalization, Contrast Limited Adaptive Histogram Equalization (CLAHE), canny edge detection, and Bilateral filtering are applied to the MRI images of the original dataset. The combined pre-processing setup has proved to be more effective in classifying PMG disorder.
2. Deep learning models such as ResNet-50, ResNet-101, VGG-16, DenseNet-201, and MobileNetV2 are tested for their ability to classify PMG images. The considered models are experimented with both original image dataset and pre-processed image dataset.
3. The GradCAM++ is adopted to analyze the areas of images being focused on by the deep learning models for the classification of PMG.

The remaining part of the article is structured as follows. The methods and materials used are discussed in Section II. Section III presents the experimental findings. Section IV gives a discussion. The conclusion is given in section V.

## Materials and methods

**Fig. 2:**
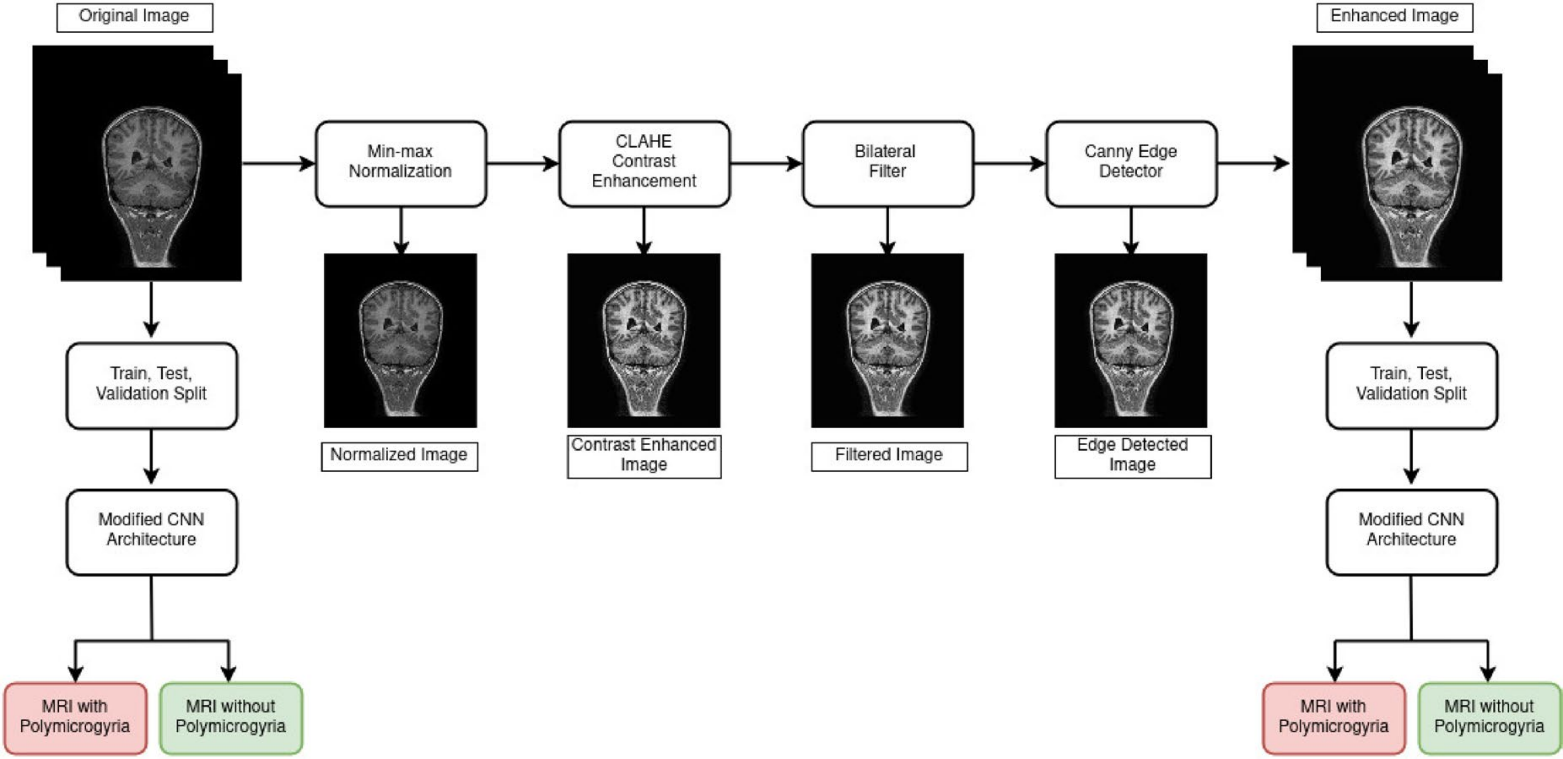
Proposed Methodology Outline.

In this research, a pediatric neurological disorder called Polymicrogyria is detected with the help of brain MRI images. Different deep-learning classifiers are trained and tested for their abilities to classify Polymicrogyria (PMG). The dataset used in the research and the proposed methodology are discussed. Figure 2 shows a general outline of our methodology.

### Dataset used

In this research, the publicly made available pediatric polymicrogyria MRI (PPMR) dataset by L. Zhang et al.^6^ is utilized. The dataset is publicly available at https://www.kaggle.com/datasets/lingfengzhang/pediatric-polymicrogyria-mri-dataset. The dataset is made up of MRI images captured from 23 patients. These MRI images contain a combination of Polymicrogyria cases and normal cases. The ratio between controls and PMG patients is 3:1. The ratio between normal cases and anomaly cases is around 5:1. On average, around 150 brain scans of each patient were included in the dataset. The patient cohort has 50% of the female population, having a mean age of 12.3 years during the scan process. The normal group consists of patients who have shown symptoms of headache and underwent MRI scans. JPEG images were exported from the images of a coronal 3D gradient echo T1 weighted sequence, which is the highest resolution sequence and best at showing cortical detail. This dataset contains 15,056 total MRI images, out of which 10,539 are control MRI images and 4517 are MRI images containing polymicrogyria.

### Image Pre-processing

Polymicrogyria (PMG) is a developmental brain disorder characterized by an abnormal increase in the number of small folds (gyri) on the brain’s surface, often appearing before birth. This condition can manifest unilaterally, affecting one side of the brain, or bilaterally, involving both sides^18,19^. The appearance of PMG on MRI scans is notably variable, influenced by several key factors. Imaging parameters such as contrast in gray and white matter regions and slice thickness can impact cortical structure visibility. The stage of brain maturity and myelination at the time of the MRI can alter the detection of subtle abnormalities^20^. The type of PMG itself plays a crucial role, as different forms can present distinct patterns in imaging. For instance, some cases may show pronounced irregularities in the cortical surface, while others might reveal more subtle changes challenging to differentiate from normal variations in brain anatomy^21,22^.

Proper image pre-processing is crucial for any algorithm to detect PMG or extract any feature under such variable conditions. All the images are converted to grayscale to reduce the color computational complexity and enhance image preprocessing techniques. This allows more effective application of techniques like histogram equalization and edge detection, enhancing the visualization of subtle tissue contrasts. Then the images are normalized using Min–Max normalization to scale the pixel intensities of the dataset images between 0–1 to ensure consistency across different MRI scans, which is essential for the model’s performance. Histogram equalization is applied to the normalized image to improve the contrast in MRI images and highlight subtle brain structure differences that are essential to detect PMG.

After obtaining an improved image, it is essential to lower the noise level without losing the quality of important anatomical features, including the cortical borders. For noise reduction, edge-preserving filters are employed. Specifically, a Bilateral Filter is used, which successfully eliminates noise while maintaining edges, protecting important anatomical features like cortical borders. Once the image’s noise has been eliminated, the Canny Edge Detector is applied for edge detection and enhancement. This step highlights small structural anomalies that are suggestive of polymicrogyria (PMG), such as irregular gyri and shallow sulci, and emphasizes cortical boundaries. This preprocessing pipeline is applied across the whole dataset, and then the enhanced dataset is fed to the model for training and inference.

### Deep learning-based models

The research aims to evaluate the performance of various deep learning models on the considered polymicrogyria (PMG) dataset. Deep learning models such as ResNet-50, ResNet-101, VGG-16, DenseNet-201, and MobileNetV2 are tested for their ability to classify PMG. The effectiveness of these models is assessed using both the original and pre-processed images to facilitate binary classification.

Due to their widespread application in both general computer vision tasks and medical imaging, the aforementioned models were chosen as representative convolutional neural networks. This ensures that the impact of the proposed preprocessing pipeline can be successfully assessed across a wide range of model complexities and architectural styles. The residual networks like ResNet-50 and ResNet-101, heavyweight models like DenseNet-201, lightweight architectures like MobileNetV2, and the traditional VGG-16 design are adopted to test the proposed methodology.

As highlighted by Xu et al.^38^, ResNet models are extensively used in medical image analysis due to their residual connections, which facilitate effective feature extraction, efficient training of deep architectures, and enhanced predictive performance. DenseNet-201 has dense connectivity that facilitates feature reuse and gradient flow. This enhances its ability to detect subtle features critical in medical diagnostics^39^. MobileNetV2 offers computational efficiency and high accuracy, making it suitable for clinical environments with limited resources^40^. VGG-16 remains a reliable baseline due to its proven ability to extract hierarchical features, particularly excelling in thyroid and brain tumor classification^41^. Together, these architectures effectively address the challenges posed by limited and imbalanced medical datasets while balancing computational demands and model performance.

Each backbone (ResNet-50, ResNet-101, VGG-16, DenseNet-201, and MobileNetV2) was loaded with ImageNet weights. The original input layer was discarded and replaced by a custom 224 × 224 × 3 input tensor, a global-average-pooling layer, and a new classifier comprising a dense layer with 256 units (ReLU, L2 = 0.001), a dropout layer (rate = 0.5) and a single-unit sigmoid output was added. All backbone layers were frozen; only this three-layer head was trainable. Models were optimized with Adam (learning rate = 0.0005, weight decay = 0.001), batch size = 32, for up to 10 epochs. Early stopping and a learning rate optimizer limited over-fitting. The dataset was split 60% / 20% / 20% for training, validation, and testing, and all images were rescaled to the 0–1 range.

### Performance evaluation metrics

The performance of the classifiers was measured using commonly employed valuation metrics such as precision, accuracy, recall, F-1 score, and area under the curve. To measure the performance of binary classification tasks in medical image classification, the true positive rate, also called recall or sensitivity, is regarded as the most important metric^23,24^. The evaluation metrics are given in Eqs. (1) to (5).

1 $$\begin{gathered} Accuracy = \frac{Number\;of\;samples\;classified\;correctly}{{Total\;number\;of\;samples}} \hfill \\ = \frac{TP + TN}{{TP + FP + TN + FN}} \hfill \\ \end{gathered}$$

2 $$\begin{aligned} {\text{Re}} call(Sensitivity) = & \frac{Number\;of\;true\;positive\;samples}{{Number\;of\;samples\;classified\;positive}} \\ & = \frac{TP}{{TP + FN}} \\ \end{aligned}$$

3 $$\begin{aligned} Specificity = & \frac{Number\;of\;true\;negayive\;samples}{{Number\;of\;samples\;classified\;negative}} \\ & = \frac{TN}{{TN + FP}} \\ \end{aligned}$$

4 $$Precision = \frac{2 \times TP}{{2 \times TP + FP + FN}}$$

5 $$F1 score = 2 \times \frac{precision*recall}{{precision + recall}}$$

### *GradCAM*++ *visualization*

Explainable AI (XAI) refers to tools and techniques that allow humans to comprehend the results produced by machine learning algorithms. In CNNs, this is generally achieved by generating visualizations such as heatmaps that emphasize regions of the image that the model gives more importance to. GradCAM++^36^ is a sophisticated visualization method employed to interpret CNNs’ decision-making process. It calculates weighted mixtures of positive partial derivatives of feature maps from the last convolutional layer relative to a specific target class. In contrast to regular GradCAM^37^, the method employs higher-order derivatives to localize multiple instances of objects more efficiently and achieve wider coverage of discriminative areas. GradCAM^++^ produces class-discriminative heatmaps that indicate areas of input images most impactful to the predictions made by the model. We have applied this visualization technique to our best-performing model to identify which areas of the MRI image the network focuses on when making classification decisions. This will improve model interpretability, model transparency, validate that the model is attending to relevant structures, and provide insights into how to guide future model improvements.

## Experiments and results

The methodology involves two types of experiments with deep learning models: one using the original image dataset and the other using a pre-processed image dataset.

### Pre-processing

The raw image is resized and converted to grayscale, which highlights the various tissue types and helps in efficient characteristic analysis without the problem of color complexity in computation. A standard gray scaling operation is used to achieve this task. Intensity normalization is applied to the grayscale image. Intensity normalization is crucial to MRI image processing because tissue properties and scanner settings might change across different MRI images. For the proposed pipeline, min–max normalization is selected. Min–max normalization scales the pixel intensities to a standard range, often between 0 and 1, without making assumptions about the data distribution. By using this normalization technique, the intrinsic intensity differences between these tissues are maintained, which is crucial for the subsequent classification tasks. To improve the visibility of subtle cortical malformations, contrast enhancement methods are applied to the normalized image. The traditional global histogram equalization technique adjusts the contrast uniformly across the image, leading to over-enhancement in some regions and under-enhancement in others. This results in the obscuration of subtle features and amplification of noise in certain areas of the image. Such noise amplification is detrimental to the detection of cortical abnormalities. Another contrast enhancement technique, Contrast Limited Adaptive Histogram Equalization (CLAHE)^25^, works by splitting the image into smaller sections and applying local histogram equalization to the regions. This makes it well-suited for improving fine details such as folding patterns and gray-white matter boundaries that are critical for PMG detection. Additionally, CLAHE includes a clip limit parameter that prevents the over-amplification of noise during contrast enhancement. Hence, CLAHE is preferred over global histogram equalization.

Noise in MRI images can distort fine anatomical details and pose a challenge to detecting subtle cortical abnormalities such as PMG. Effective noise reduction is essential for enhancing the images without compromising the critical structures like cortical thickness and the shape of gyri and sulci. Several noise reduction filters were explored for this purpose, and each was evaluated based on its ability to preserve important anatomical features while smoothening noise. The first procedure employed was to use a Bilateral filter^26^ to filter out the noise while preserving the details of edges. This non-linear filter implements a weighted averaging process where each pixel’s new value is computed by examining neighboring pixels within a defined spatial window. For each neighboring pixel, two weights are calculated: a spatial weight based on geometric distance and a range weight based on intensity similarity. The final filtered value is the weighted sum of neighboring intensities, where pixels that are both spatially close and intensity-similar have the highest influence. This dual-weighting mechanism effectively removes high-frequency noise while preserving cortical boundaries essential for PMG detection.

Another method employed was to use a Non-Local Means^27^ filter to denoise the patches of similar texture or intensity across the image, instead of considering just the local neighborhoods. The method is effective in preserving fine structural details and textures, which is important to detect PMG. While this method excels in noise reduction and detail preservation, its high computational cost, due to the large number of comparisons made for each pixel, makes it less applicable for large-scale and real-time use in PMG detection.

Anisotropic Diffusion^28^ method is utilized to reduce noise by diffusing intensity along gradients. The method has been widely used for medical images to maintain complex anatomical structures, such as boundaries between gray and white matter. Anisotropic Diffusion requires careful tuning of parameters, which makes optimization challenging across image variations. This limitation is problematic for PMG detection, as polymicrogyria presents diverse structural variations. Thus, despite the effectiveness, the limitations make it less desirable for PMG detection compared to other methods.

A procedure called Wavelet Denoising technique^29,30^, is also employed which reduces noise by decomposing images into different frequency bands and selectively reducing noise in the high-frequency components. This approach is advantageous for preserving the fine details and large structures in MRI images, making it a promising option for PMG detection. The method requires careful selection of wavelength thresholds, which can complicate the implementation when dealing with subtle and highly variable cortical abnormalities. This complexity involved in tuning wavelength parameters makes it less suitable as compared to other methods.

Among the considered filters, the Bilateral filter maintains the critical anatomical details in the image with reduced complexities in parameter selection and computation. Hence the Bilateral Filter was chosen as the best option as it provided an optimal balance between noise suppression and edge preservation. After the reduction of noise, an attempt was made to enhance the edges in the image using different edge detection techniques. Several methods such as Canny edge detector, Scharr Operator, Sobel operator, and Laplacian of Gaussian, were tested on the filtered image. The canny edge detector performed better than the other methods as it identified significant edges while minimizing the detection of noise-induced spurious edges. Canny’s multi-stage process comprising noise reduction, non-maximum suppression, gradient calculation, and edge tracking ensured that only the most relevant or strong edges were detected. This is important in MRI images as precise edges are crucial for detecting subtle cortical irregularities associated with PMG. The Sobel and Scharr operators were prone to amplifying noise and failed to detect fine cortical abnormalities effectively. Laplacian of Gaussian was found to blur some of the fine cortical details necessary for accurate PMG detection. The Canny edge detector with its superior edge localization and noise handling capabilities was the most reliable method to enhance the edges.

**Fig. 3:**
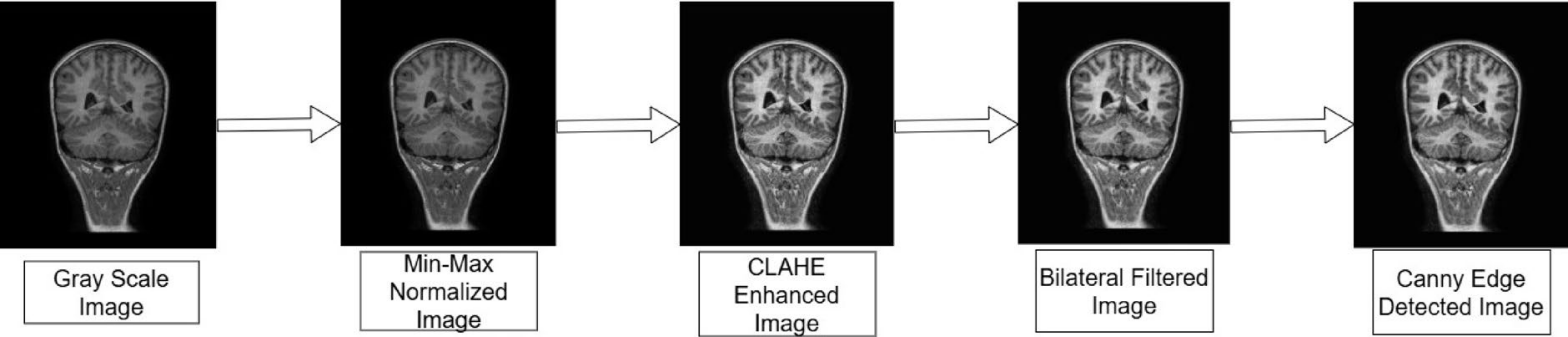
Pre-processing Pipeline.

The proposed image pre-processing approach integrates the Min–Max normalization, CLAHE for contrast enhancement, Bilateral filtering for efficient noise reduction, and Canny edge detection to prepare the MRI image dataset for the detection of PMG. This sequence of techniques was found to provide the best balance in preserving critical anatomical details as well as reducing noise while enhancing local contrast and detecting relevant edges. The image pre-processing involved applying min–max normalization to scale pixel intensities, followed by contrast enhancement using CLAHE with a clip limit of 2.0 and a tile grid size of 8 by 8. Noise reduction was performed using a bilateral filter with a kernel diameter of 9 and sigma values of 75 for both color and space. Edge enhancement utilized the Canny edge detector with thresholds of 50 and 200, an aperture size of 3, and the edge map was blended at an alpha value of 0.20. The summary of the image pre-processing approach adopted in this research is illustrated in Fig. 3.

### Classification using original images

The considered dataset comprises 15,056 MRI images, with 10,539 being controls and 4,517 containing polymicrogyria. This imbalance results in a skewed distribution, leading to overfitting during training. Models like ResNet-50, VGG-16, and others achieve high training accuracy (97–100%) but exhibit much lower validation accuracy (around 30%). This discrepancy indicates that the networks are not generalizing well and are biased towards the majority class. To resolve this issue, the dataset was balanced by selecting an equal number of control and polymicrogyria images, resulting in 4,517 images per class. The control images were selected using random sampling to maintain an unbiased representation of standard brain MRI patterns. The authors have considered down-sampling the control class rather than augmenting the polymicrogyria (PMG) class, as PMG exhibits subtle and complex cortical variations that are crucial for diagnosis. The traditional augmentation methods, like rotation or scaling, could distort these essential features. The goal of the research is to assess the effect of the proposed pre-processing pipeline on the original images. Adoption of augmentation could introduce artificial variability that might obscure the genuine effects of the pre-processing steps. This approach helps prevent overfitting and enhances the models’ generalization capabilities due to the balanced class distribution.

Pre-trained models such as ResNet-50, ResNet-101, VGG-16, MobileNetV2, and DenseNet-201 are adopted for the classification^31–34^. Each model’s base layers were frozen to retain the features learned from ImageNet^35^, which were trained for general image classification tasks. Fully connected dense layers were added on top of the frozen layers. only these additional layers were trained to fine-tune the models for the binary classification task without losing the pre-trained knowledge. The MRI images were converted to RGB format and resized to 224 × 224 pixels to match the input size and color mode required by these models, which are pre-trained on ImageNet.

**Fig. 4:**
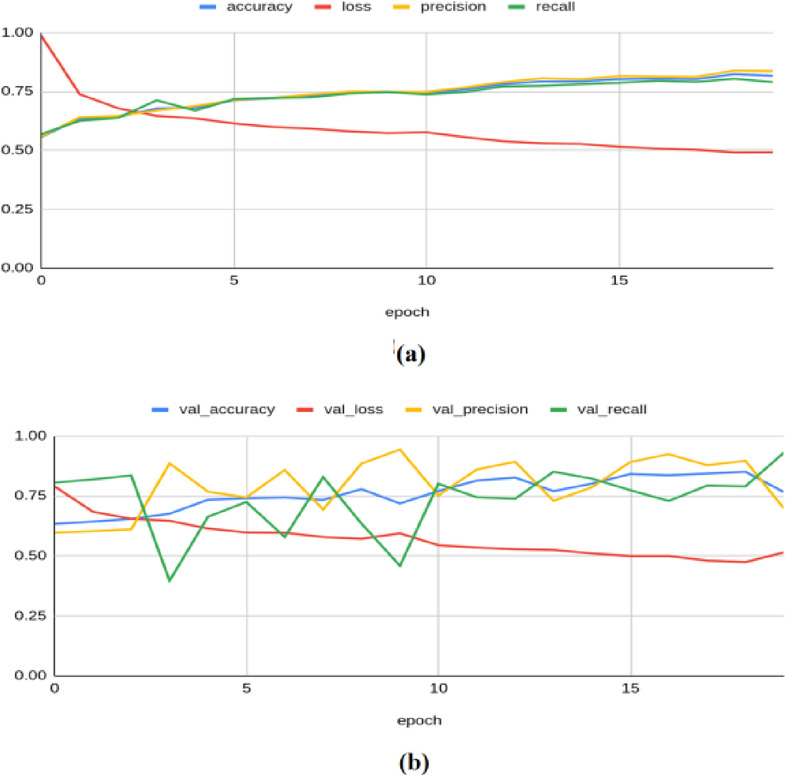
Classification results produced by ResNet-50 architecture: (**a**) Training Results (**b**) Validation Results.

To illustrate the convergence and overfitting of models, the accuracy and loss curves are more appropriate. As demonstrated in^42^, such visualizations significantly enhance the transparency of the model training process, and hence, these plots are adopted to illustrate the results. The Resnet-50 model achieved an accuracy of 0.8155, a loss of 0.4908, a precision of 0.8361, a recall of 0.7883, an F1 score of 0.751, and a Cohen’s kappa value of 0.603 on the training set. On the validation set, the model yielded an accuracy of 0.7654, a loss of 0.5137, a precision of 0.6974, a recall of 0.9296, an F1 score of 0.783, and kappa value of 0.614 as illustrated in Fig. 4. On the test set, the model produced an accuracy of 0.8367, a precision of 0.8764, a recall of 0.7791, a loss of 0.484, an F1 score of 0.793, and a Kappa value of 0.640.

**Fig. 5:**
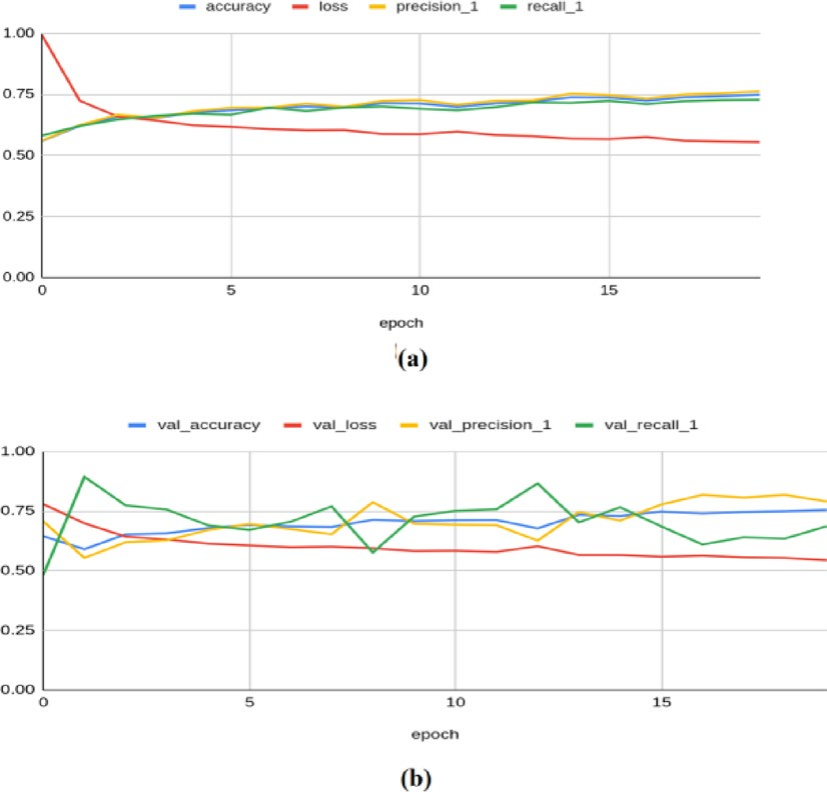
Classification results produced by ResNet-101 architecture: (**a**) Training Results (**b**) Validation Results.

The ResNet-101 model has produced a training accuracy of 0.748, along with a loss of 0.554, a precision of 0.761, a recall of 0.727, an F1 score of 0.683, and a Kappa value of 0.422. On the validation set, it yielded an accuracy of 0.754, a loss of 0.543, a precision of 0.790, a recall of 0.685, an F1 score of 0.722, and a Kappa value of 0.423, as shown in Fig. 5. For the test set, the model produced an accuracy of 0.7515, a precision of 0.7895, a recall of 0.6771, a loss of 0.55, an F1 score of 0.718, and a Kappa value of 0.470.

**Fig. 6:**
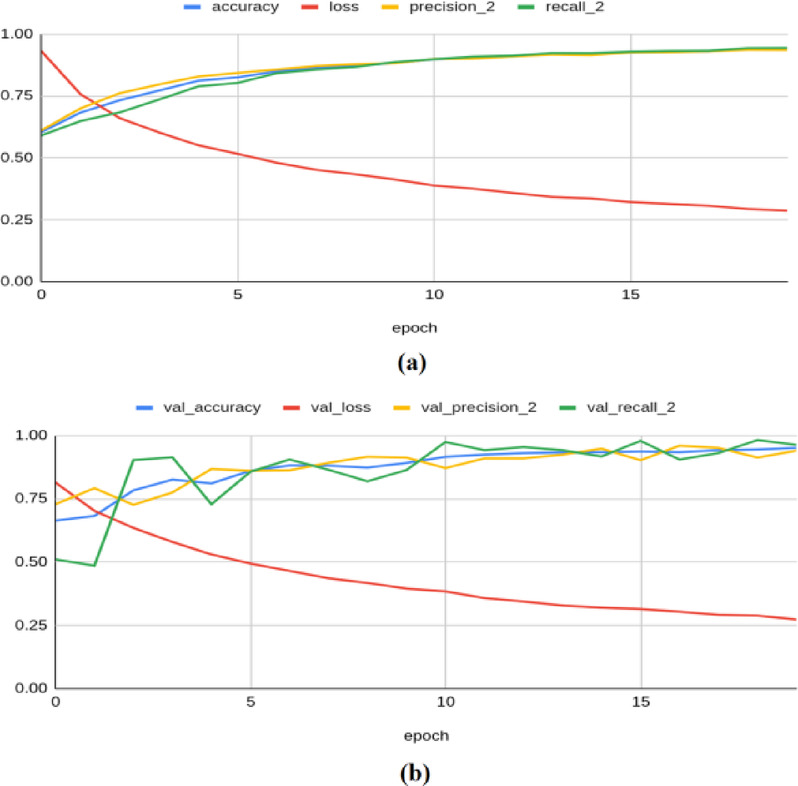
Classification results produced by VGG-16 architecture: (**a**) Training Results (**b**) Validation Results.

The VGG-16 architecture has produced a training accuracy of 0.938, a loss of 0.286, a precision of 0.934, a recall of 0.943, an F1 score of 0.929, and a Kappa value of 0.904. On the validation set, it yielded an accuracy of 0.950, a loss of 0.272, a precision of 0.939, a recall of 0.962, an F1 score of 0.950, and a Kappa value of 0.899, as shown in Fig. 6. For the test set, the model produced an accuracy of 0.9513, a precision of 0.9447, a recall of 0.9574, a loss of 0.2772, an F1 score of 0.949, and a Kappa value of 0.897.

**Fig. 7:**
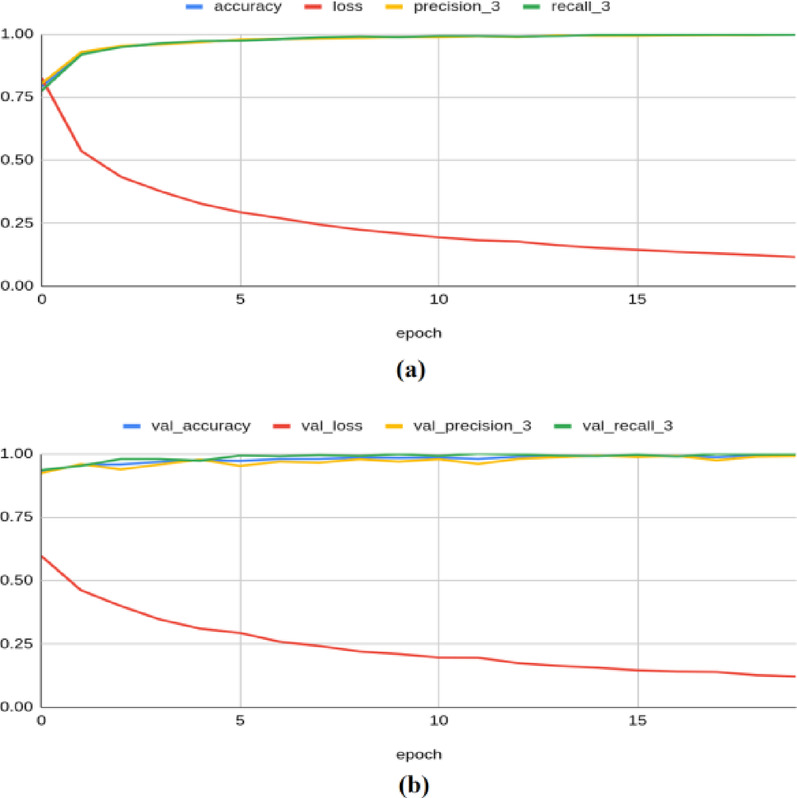
Classification results produced by MobileNetV2 architecture: (**a**) Training Results (**b**) Validation Results.

The MobileNetV2 framework achieved a training accuracy of 0.997, a loss of 0.115, a precision of 0.998, a recall of 0.997, an F1 score of 0.989, and a Kappa value of 0.994. On the validation set, it yielded an accuracy of 0.994, a loss of 0.121, a precision of 0.990, a recall of 0.998, an F1 score of 0.976, and a Kappa value of 0.980, as shown in Fig. 7. For the test set, the model produced an accuracy of 0.9889, a precision of 0.9921, a recall of 0.9854, a loss of 0.1282, an F1 score of 0.991, and a Kappa value of 0.983.

**Fig. 8:**
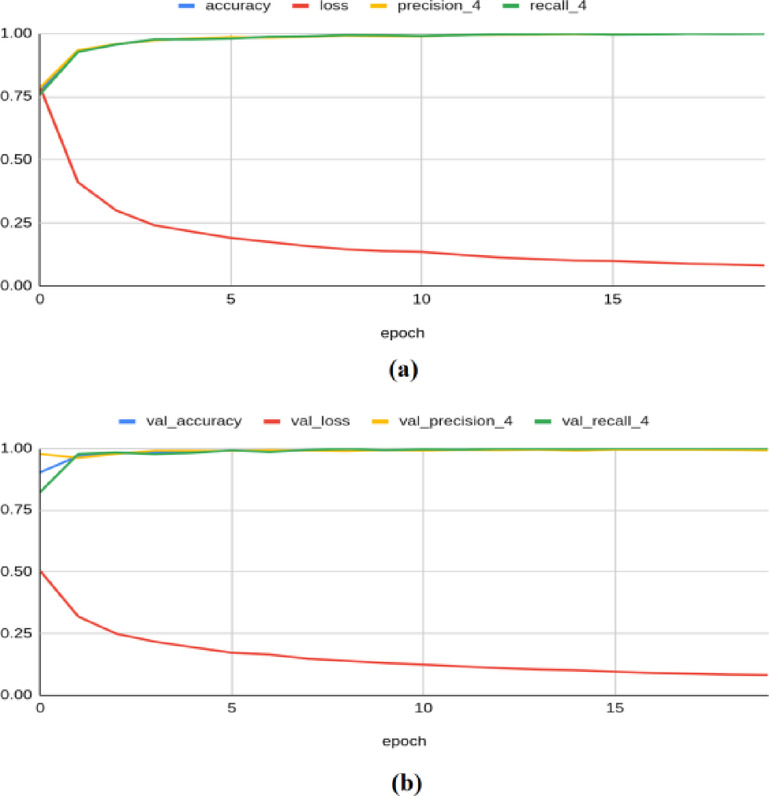
Classification results produced by DenseNet-201 architecture: (**a**) Training Results (**b**) Validation Results.

The DenseNet-201 model achieved a training accuracy of 0.998, a loss of 0.081, a precision of 0.998, a recall of 0.998, an F1 score of 0.992, and a Kappa value of 0.998. On the validation set, it yielded an accuracy of 0.995, a loss of 0.081, a precision of 0.991, a recall of 0.999, an F1 score of 0.996, and a Kappa value of 0.992, as shown in Fig. 8. For the test set, the model produced an accuracy of 0.9967, a precision of 0.9933, a recall of 1.000, a loss of 0.0803, an F1 score of 0.993, and a Kappa value of 0.987.

The summary of the results obtained by all classifiers is presented in Table 2. As observed, DenseNet-201 delivers the best performance, achieving high values for accuracy, precision, and recall across all datasets, with the lowest loss compared to other models.

**Table 2:** Classification results on the original pmg dataset.

Dataset	Metric	ResNet-50	ResNet-101	VGG-16	MobileNetV2	DenseNet-201
Training	Accuracy	0.815	0.747	0.938	0.997	0.998
	Loss	0.490	0.554	0.285	0.114	0.080
	Precision	0.836	0.761	0.934	0.998	0.998
	Recall	0.788	0.726	0.943	0.996	0.998
	F1 Score	0.751	0.683	0.929	0.989	0.992
	Cohen’s Kappa	0.603	0.422	0.904	0.994	0.998
Validation	Accuracy	0.765	0.753	0.950	0.993	0.995
	Loss	0.513	0.543	0.272	0.120	0.081
	Precision	0.697	0.789	0.938	0.990	0.991
	Recall	0.929	0.684	0.962	0.997	0.998
	F1 Score	0.783	0.712	0.950	0.976	0.996
	Cohen’s Kappa	0.614	0.423	0.899	0.980	0.992
Test	Accuracy	0.836	0.751	0.951	0.988	0.996
	Loss	0.484	0.551	0.277	0.128	0.080
	Precision	0.876	0.789	0.944	0.992	0.993
	Recall	0.779	0.677	0.957	0.985	0.998
	F1 Score	0.793	0.708	0.949	0.991	0.993
	Cohen’s Kappa	0.640	0.470	0.897	0.983	0.987

To further validate the model’s performance and ensure reproducibility across different data partitions, a fivefold cross-validation analysis was conducted on the considered dataset. The cross-validation results are presented in Table 3, where the ± values represent the standard deviation of validation losses across the five folds, indicating the consistency of model performance across different data partitions. The relatively small standard deviations demonstrate stable model behavior and consistent learning patterns, mitigating concerns about performance variability due to random data partitioning. The K-fold cross-validation approach yielded substantially improved performance compared to the initial 60-20-20 split methodology, particularly benefiting the deeper ResNet architectures. This improvement can be attributed to the increased training data utilization inherent in the K-fold approach, while the original split used only 60% of the dataset for training, each fold in the fivefold cross-validation utilized 80% of the data for training, representing a 33% increase in training samples.

**Table 3:** Classification results on the original PMG dataset using the K-Fold method.

Architecture	Test Accuracy (%)	Test Precision (%)	Test Recall (%)	Mean Validation Loss	Std Dev ( ±)
VGG-16	100.00	100.00	100.00	0.0020	0.0009
MobileNetV2	98.12	98.01	98.23	0.0882	0.0109
DenseNet-201	97.68	99.09	96.23	0.0793	0.0078
ResNet-50	96.02	96.32	95.68	0.1361	0.0203
ResNet-101	95.68	95.68	95.68	0.1519	0.0380

### Classification using pre-processed images

The considered models are also experimented with the pre-processed image dataset while retaining the same experimental setup used for the original images. The models’ performances on the pre-processed image datasets are also measured in accuracy, precision, recall, and loss.

**Fig. 9:**
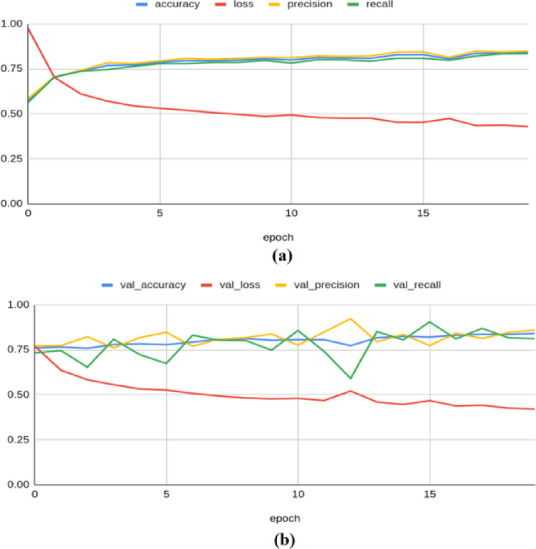
Classification results produced by Resnet-50 architecture: (**a**) Training Results (**b**) Validation Results.

The Resnet-50 model achieved an accuracy of 0.841, a loss of 0.429, a precision of 0.848, a recall of 0.833, an F1 score of 0.794, and a Kappa value of 0.640 on the training set. On the validation set, it yielded an accuracy of 0.840, a loss of 0.420, a precision of 0.858, a recall of 0.811, an F1 score of 0.830, and a Kappa value of 0.656 as shown in Fig. 9. For the test set, the model produced an accuracy of 0.8489, a precision of 0.8689, a recall of 0.8173, a loss of 0.4191, an F1 score of 0.839, and a Kappa value of 0.673.

**Fig. 10:**
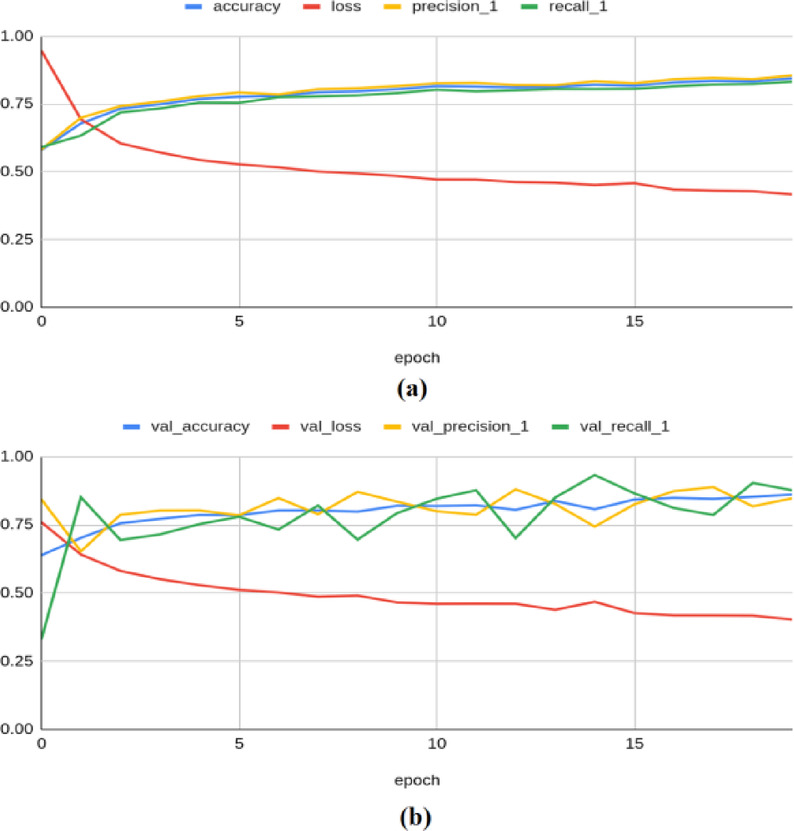
Classification results produced by Resnet-101 architecture: (**a**) Training Results (**b**) Validation Results.

The Resnet-101 architecture achieved a training accuracy of 0.844, a loss of 0.416, a precision of 0.855, a recall of 0.832, an F1 score of 0.806, and a Kappa value of 0.652. On the validation set, it yielded an accuracy of 0.860, a loss of 0.402, a precision of 0.847, a recall of 0.876, an F1 score of 0.829, and a Kappa value of 0.665 as illustrated in Fig. 10. For the test set, the model produced an accuracy of 0.8544, a precision of 0.8452, a recall measure of 0.8632, a loss of 0.4040, an F1 score of 0.844, and a Kappa value of 0.674.

**Fig. 11:**
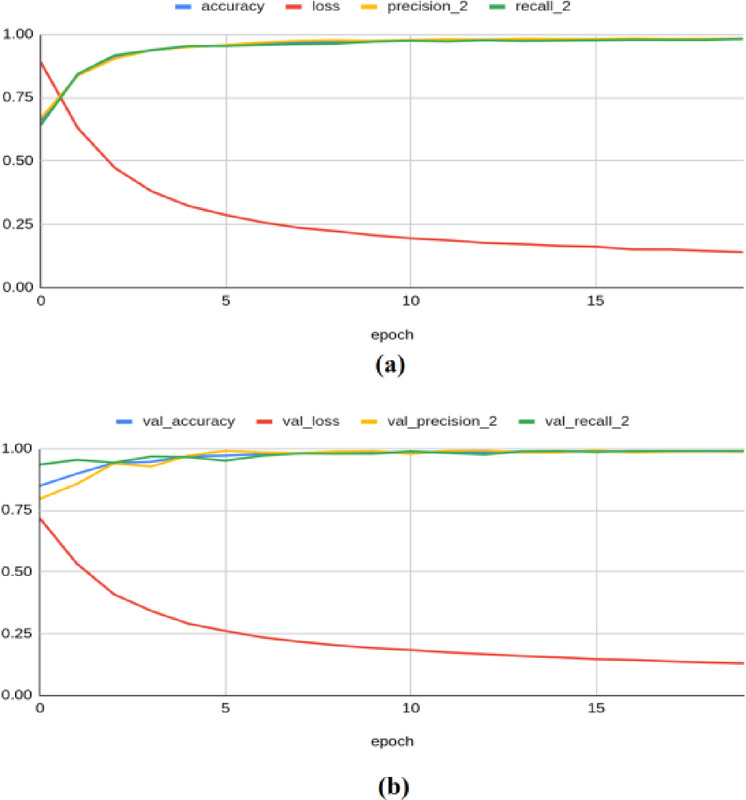
Classification results produced by VGG-16 architecture: (**a**) Training Results (**b**) Validation Results.

The VGG-16 model achieved a training accuracy of 0.981, a loss of 0.139, a precision of 0.981, a recall of 0.981, an F1 score of 0.980, and a Kappa value of 0.966. On the validation set, it yielded an accuracy of 0.988, a loss of 0.128, a precision of 0.987, a recall of 0.989, an F1 score of 0.983, and a Kappa value of 0.968 as shown in Fig. 11. For the test set, the model produced an accuracy of 0.9856, a precision of 0.9876, a recall of 0.9832, a loss of 0.1342, an F1 score of 0.984, and a Kappa value of 0.969.

**Fig. 12:**
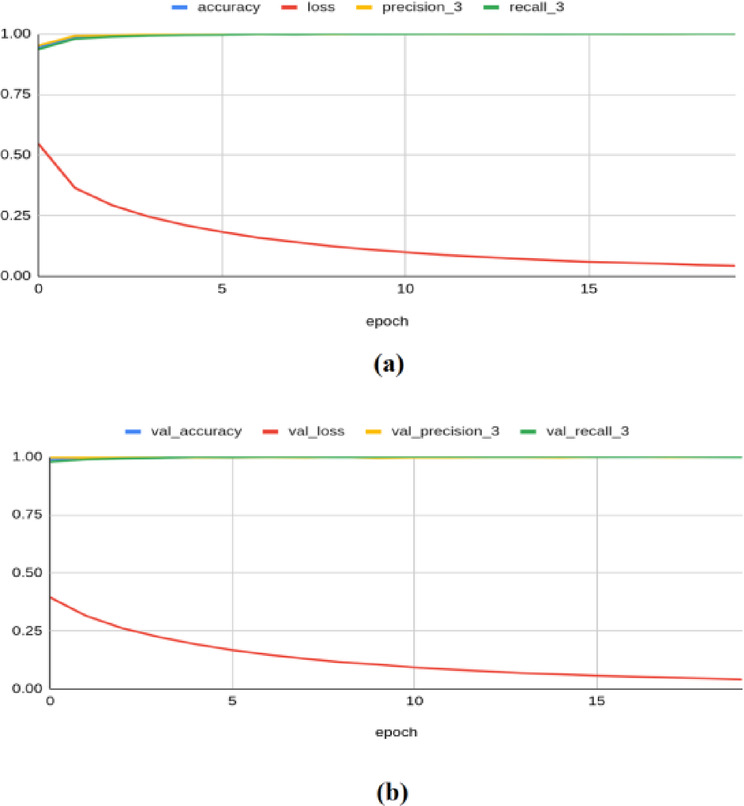
Classification results produced by MobileNetV2 architecture: (**a**) Training Results (**b**) Validation Results.

The MobileNetV2 model achieved a training accuracy of 0.999, a loss of 0.042, a precision of 0.999, a recall of 0.999, an F1 score of 0.996, and a Kappa value of 0.999. On the validation set, it yielded an accuracy of 0.999, a loss of 0.040, a precision of 1.000, a recall of 0.998, an F1 score of 0.998, and a Kappa value of 0.996 as shown in Fig. 12. For the test set, the model produced an accuracy of 0.9983, a precision of 1.0000, a recall of 0.9966, a loss of 0.0414, an F1 score of 0.999, and a Kappa value of 0.998.

**Fig. 13:**
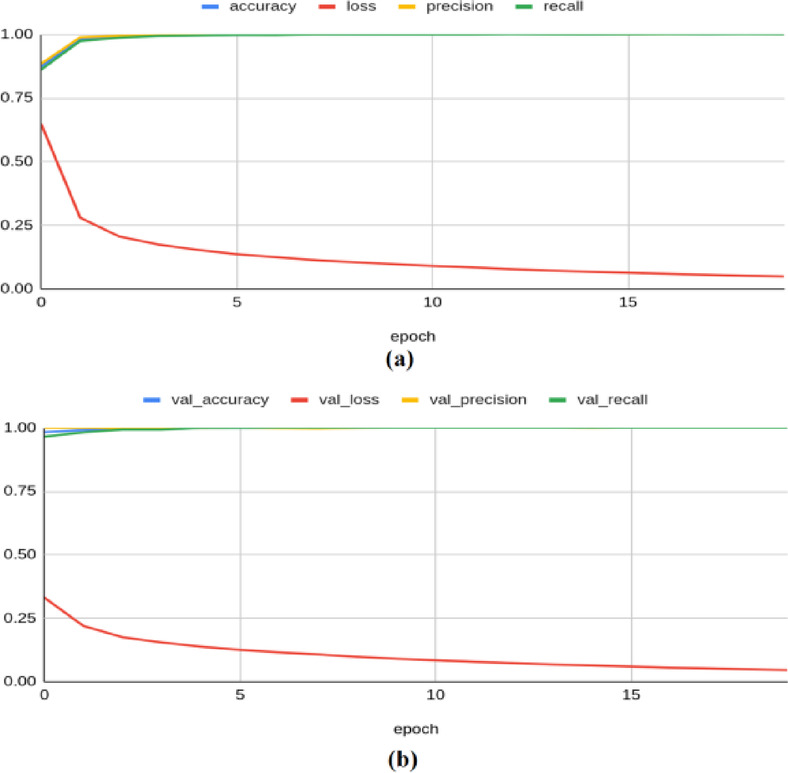
Classification results produced by VGG-16 architecture: (**a**) Training Results (**b**) Validation Results.

The Densenet-201 framework achieved a training accuracy of 0.999, a loss of 0.048, a precision of 0.999, a recall of 0.999, an F1 score of 0.995, and a Kappa value of 1.000. On the validation set, it yielded an accuracy of 1.000, a loss of 0.045, a precision of 1.000, a recall of 1.000, an F1 score of 0.999, and a Kappa value of 0.997, as shown in Fig. 13. It also produced a testing accuracy of 1.0, a precision of 1.0, a recall value of 1.0, a loss of 0.0467, an F1 score of 0.999, and a Kappa value of 0.999 on the test set.

The summary of the results obtained by all classifiers on the pre-processed PMG image dataset is presented in Table 4. DenseNet-201 produces a better output when compared to the other models due to its high performance on the test set.

**Table 4:** Classification results on the pre-processed pmg dataset.

Dataset	Metric	ResNet-50	ResNet-101	VGG-16	MobileNetV2	DenseNet-201
Training	Accuracy	0.840	0.843	0.980	0.999	0.999
	Loss	0.429	0.415	0.139	0.042	0.048
	Precision	0.847	0.854	0.981	0.999	0.999
	Recall	0.833	0.831	0.980	0.999	0.999
	F1 Score	0.794	0.806	0.980	0.996	0.995
	Cohen’s Kappa	0.640	0.652	0.966	0.999	1.000
Validation	Accuracy	0.840	0.859	0.987	0.998	1.000
	Loss	0.419	0.401	0.128	0.040	0.045
	Precision	0.858	0.846	0.986	1.000	1.000
	Recall	0.811	0.875	0.988	0.997	1.000
	F1 Score	0.830	0.829	0.983	0.998	0.999
	Cohen’s Kappa	0.656	0.665	0.968	0.996	0.997
Test	Accuracy	0.848	0.854	0.985	0.998	1.000
	Loss	0.419	0.404	0.134	0.041	0.046
	Precision	0.868	0.845	0.987	0.996	1.000
	Recall	0.817	0.863	0.983	1.000	1.000
	F1 Score	0.839	0.844	0.984	0.999	0.999
	Cohen’s Kappa	0.673	0.674	0.969	0.998	0.999

As mentioned in the previous section, a fivefold cross-validation is performed in addition to the original train-validation-test split approach. The cross-validation results demonstrated remarkable consistency across all architectures, as shown in Table 5. The ± values represent the standard deviation across the five folds, indicating the variability in performance. The consistently low standard deviations demonstrate robust generalizability despite the limited dataset size, strongly supporting the reliability of the proposed approach. As also mentioned in the previous section, the K-fold method yielded substantial performance improvement over the train-test-split approach. But, as seen in Table 6, the use of pre-processed images yields a significant improvement compared to using the original images.

**Table 5:** Classification results on the pre-processed pmg dataset using the K-fold method.

Architecture	Test Accuracy (%)	Test Precision (%)	Test Recall (%)	Mean Validation Loss	Std Dev ( ±)
VGG-16	100.00	100.00	100.00	0.0010	0.0004
DenseNet-201	99.94	99.89	100.00	0.0050	0.0011
ResNet-101	99.67	99.78	99.56	0.0157	0.0018
ResNet-50	99.61	100.00	99.22	0.0183	0.0037
MobileNetV2	99.50	99.23	99.78	0.0110	0.0024

**Table 6:** Comparison of K-Fold results on the original and pre-processed image.

Architecture	Original Images			Enhanced Images			Performance Improvements		
	Accuracy (%)	Precision (%)	Recall (%)	Accuracy (%)	Precision (%)	Recall (%)	Accuracy (+ %)	Precision (+ %)	Recall (+ %)
VGG-16	100.00	100.00	100.00	100.00	100.00	100.00	0.00	0.00	0.00
MobileNetV2	98.12	98.01	98.23	99.50	99.23	99.78	+ 1.38	+ 1.22	+ 1.55
DenseNet-201	97.68	99.09	96.23	99.94	99.89	100.00	+ 2.26	+ 0.80	+ 3.77
ResNet-50	96.02	96.32	95.68	99.61	100.00	99.22	+ 3.59	+ 3.68	+ 3.54
ResNet-101	95.68	95.68	95.68	99.67	99.78	99.56	+ 3.99	+ 4.10	+ 3.88

### Statistical testing

To evaluate the statistical significance of performance differences between independently retrained models on normal and enhanced images, McNemar’s test and bootstrap analysis were employed. McNemar’s test is a well-established method for comparing the sensitivities and specificities of two diagnostic techniques administered to the same group of patients, focusing on discordant pairs where the tests disagree to identify significant variations in diagnostic performance^43,44^. Bootstrap analysis with 1000 iterations was used to generate confidence intervals for key metrics, providing robust non-parametric estimates particularly suitable for medical imaging datasets with limited sample sizes^45^.

The McNemar produced a chi-square statistic of 29.47 (p < 0.001), indicating significant differences in model predictions on the same test cases. The model trained on enhanced images correctly identified 44 cases that the model trained on normal images misclassified. It only failed on 5 cases where the normal model succeeded. This demonstrates better diagnostic ability.

The preprocessing pipeline achieved significant improvements across all performance metrics. Accuracy increased by 2.16%, indicating better overall diagnostic correctness. Precision improved by 1.61%, meaning fewer false positive diagnoses that could lead to unnecessary interventions. Recall increased by 2.80%, resulting in fewer missed positive cases, which is crucial for early disease detection. The F1-score improved by 2.21%, showing better balanced performance between precision and recall. The AUC also increased by 0.27%, demonstrating a better ability to distinguish between disease classes across all threshold values.

Bootstrap confidence intervals confirmed the reliability of these gains. The intervals did not overlap for both accuracy (Normal: 96.5%-97.9% vs Enhanced: 99.0%-99.7%) and AUC (Normal: 99.6%-99.8% vs Enhanced: 99.97%-100%). This suggests significant and reproducible gains in performance. The results of statistical tests are given in Table 7.

**Table 7:** Statistical results of the Densenet-201 model trained on normal and enhanced images.

Metric	Normal Model	Enhanced Model	Difference between enhanced and normal	Confidence Interval — Normal	Confidence Interval — Enhanced
Accuracy	0.9723	0.9939	0.0216	0.9646 – 0.9790	0.9900 – 0.9972
Precision	0.9828	0.9989	0.0161	—	—
Recall	0.9608	0.9888	0.028	—	—
F1-Score	0.9717	0.9938	0.0221	—	—
AUC	0.9972	0.9998	0.0027	0.9958 – 0.9984	0.9997 – 1.0000
Loss	0.093	0.0233	–0.0698	—	—

### Ablation study

As demonstrated by the results, the model is well generalized and consistent. It is also evident that the pre-processing pipeline produces enhanced results. To check the impact that each step has on the model accuracy, an ablation study is performed using the best-performing model, DenseNet201.

The results, as shown in Table 8, demonstrate clear incremental improvements with each additional preprocessing step. Notably, grayscale conversion provided no performance improvement over the original images, maintaining identical metrics across all evaluation criteria. Despite having no performance gain, this step is crucial for computational efficiency, as it reduces processing overhead and memory requirements of the subsequent operations. Normalization provides the first improvement, increasing the accuracy from 80.6% to 81.68% and the F1 score from 0.83 to 0.84. Subsequently, the application of CLAHE and then bilateral filtering improves the model performance, resulting in a 96.96% accuracy. The final step of Canny edge detection maintains the same high accuracy of 96.96%, but achieves important optimization benefits, which include lower test loss, improved recall, and a marginally better F1 score. The progressive improvement from 80.80% to 96.96% accuracy demonstrates a 16.16-point gain, validating that the preprocessing pipeline improves model performance and that each step has a unique and direct impact on the performance.

**Table 8:** Ablation Study Results.

Pipeline	Test Loss	Test Accuracy	Test Precision	Test Recall	Test F1
Original Images	0.3759	0.8080	0.7249	0.9922	0.8378
Grayscale Conversion	0.3759	0.8080	0.7249	0.9922	0.8378
Grayscale + Normalization	0.3683	0.8168	0.7344	0.9922	0.8441
Grayscale + Normalization + CLAHE	0.2768	0.8932	0.8293	0.9900	0.9026
Grayscale + Normalization + CLAHE + Bilateral Filtering	0.1229	0.9696	0.9680	0.9712	0.9696
Full Pipeline (Includes Edges Blend)	0.1183	0.9696	0.9569	0.9834	0.9700

### *GradCam*++ *results*

**Fig. 14:**
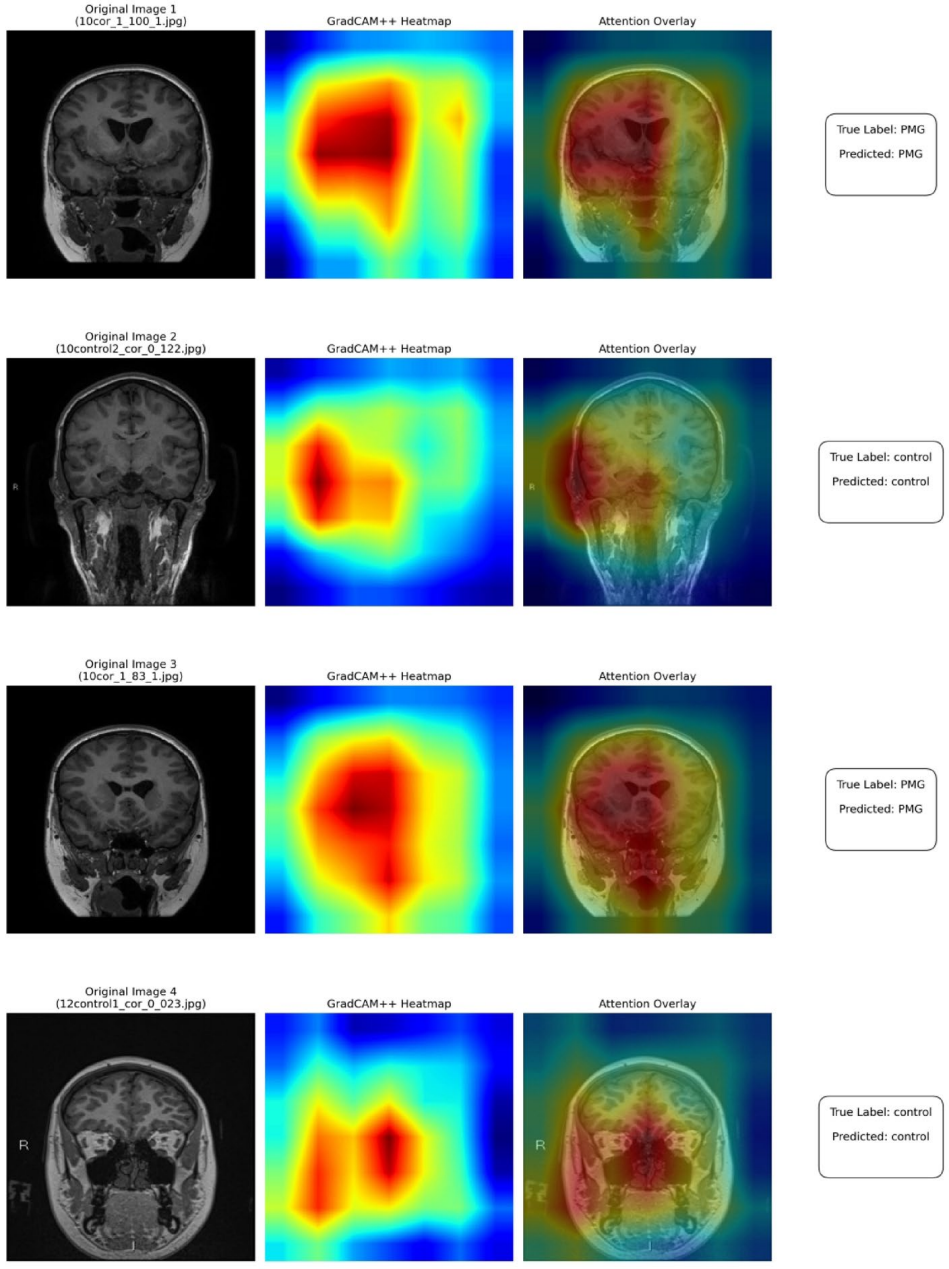
GradCam++ Heatmaps.

As demonstrated by the results, it is evident that DenseNet-201 trained on the enhanced images provides us with the highest accuracy. On applying GradCam++ on this model, visualizations that provided valuable insights into the decision-making process of the CNN model were achieved. Figure 14 illustrates the visualization results across samples from both PMG and control classes.

For PMG cases, the model focused on cortical areas where the brain gyri were asymmetrical, with structures that are characteristic of polymicrogyria. Control cases, on the other hand, had more scattered attention patterns, with activation spread across several brain areas instead of intensely focusing on individual gyral formations. These attention maps validate the fact that the model focuses on anatomically appropriate structures, improving model transparency and reliability.

Although IoU could not be calculated because the dataset lacks radiologist-drawn lesion masks, we conducted a quantitative assessment of GradCAM++ heatmaps on a representative subset of PMG and control images. Eight summary statistics were extracted from each activation map, including activation strength (mean, maximum, standard deviation), distribution measures (entropy, Gini coefficient), thresholded area ratios, total spread, and center-of-mass distance. PMG images consistently showed stronger and more concentrated activations compared to controls across all metrics (mean activation: 0.503 ± 0.035 vs 0.456 ± 0.007; activation ratio ≥ 0.5: 0.478 ± 0.086 vs 0.405 ± 0.031, where ± represents standard deviation), with large effect sizes (Cohen’s d ≈ 1–2.6). These quantitative findings support the visual observations that the model focuses on anatomically relevant cortical regions for PMG classification.

## Discussion

The performance of the considered models on both the original image dataset and the processed dataset is assessed based on the performance metrics. The comparison gives a substantial insight into the effect of image pre-processing on MRI image classification. The findings demonstrate that pre-processing facilitates more efficient feature extraction by CNNs, leading to enhanced classification accuracy across all models during testing. Performance improvement is particularly noticeable in the ResNet-50, ResNet-101, and VGG-16 architectures. The pre-processing significantly improved model performance across all architectures. For instance, ResNet-50’s training accuracy improved from 81.55% to 84.08%, an improvement of 2.53%. Similarly, the test accuracy has improved from 83.67% to 84.89%, an improvement of 1.22%. ResNet-101 experienced a notable increase in training accuracy of 9.63%, from 74.76% to 84.39%. The test accuracy has been improved from 75.15% to 85.45%, with an increase of 10.3%. VGG-16’s training accuracy ascended from 93.80% to 98.10%, with an enhancement of 4.30%. The test accuracy has improved from 95.13% to 98.56%, with an improvement of 3.43%. The comparison of training accuracies on the original and pre-processed datasets is given in Table 9. The comparison of test accuracies on the original and pre-processed datasets is given in Table 10. These advancements suggest that pre-processing techniques effectively unveil pertinent features of the images while mitigating noise, thereby enabling the CNNs to learn more discriminative representations. It is noteworthy that while MobileNetV2 and DenseNet-201 also exhibited improvements, the gains were comparatively modest.

**Table 9:** Comparison of training accuracies on the datasets.

	ResNet-50	ResNet-101	VGG-16	MobileNetV2	DenseNet-201
Original dataset	81.55%	74.76%	93.80%	99.70%	99.80%
Pre-processed dataset	84.08%	84.39%	98.10%	99.90%	99.90%
Improvement	2.53%	9.63%	4.3%	0.2%	0.1%

**Table 10:** Comparison of test accuracies on the datasets.

	ResNet-50	ResNet-101	VGG-16	MobileNetV2	DenseNet-201
Original dataset	83.67%	75.15%	95.13%	98.80%	99.60%
Pre-processed dataset	84.89%	85.45%	98.56%	99.80%	100%
Improvement	1.22%	10.3%	3.43%	1.0%	0.4%

Despite these gains, several methodological and ethical limitations are present in the study. This study was conducted on a single dataset from one institution, meaning that results may vary significantly when applied to other datasets due to the specific parameter configuration of the proposed preprocessing pipeline, which was optimized for this particular dataset. The models employed (ResNet-50, ResNet-101, VGG-16, DenseNet-201, and MobileNetV2) are general-purpose architectures originally designed for natural image classification; a model specifically designed for polymicrogyria detection may outperform all tested architectures with or without preprocessing steps. While GradCAM++ visualization provides insight into where the model focuses during decision-making, thereby increasing trust and interpretability, the absence of radiologist markings or ground-truth region annotations prevents us from definitively confirming that the model focuses on anatomically correct or diagnostically relevant features. From a clinical perspective, the pipeline may function solely as decision support, as diagnostic errors could have serious consequences.

### Comparative study

We compare the proposed methodology with other relevant existing works in the literature. Table 11 gives a comparison of the obtained results. There are a few studies that focus on applying machine learning to MRI images for the classification of polymicrogyria (PMG). Hence, we have considered relevant and allied works for the comparative study.

**Table 11:** Comparative study table.

Reference	Datasets Used	Features or Techniques	Classifiers	Results Reported (%)
^6^	PPMR dataset	CDCM loss function	ResNet50	Recall—88.07, Precision – 71.86
^7^	Embryonic brain dataset	Deep features	SVM	Accuracy – 87.7
^4^	Training – Internal dataset. Testing – National Institute of Health (NIH) pediatric brain MRI database and the Developing Human Connectome Project (dHCP) database	Combining 2D and 3D CNN into an ensemble to predict myelin maturation age	3D CNN from^46^ and EfficientNet-b0 as the 2D CNN	MAE Results: Cross-validation set: 2D model – 1.53, 3D model – 2.06, Ensemble model – 1.63 Internal test set: 2D model – 1.43, 3D model – 2.55, Ensemble model – 1.77 External NIH dataset: 2D model – 2.26, 3D model – 2.27, Ensemble model – 1.22 External dHCP dataset: 2D model – 0.44, 3D model – 0.27, Ensemble model – 0.31
^32^	Publicly available Brain Tumor MRI dataset. These images were classified into two classes: images with or without a tumor	Transfer learning to evaluate and compare multiple pre-trained deep learning models	VGG-16, Inception-v3, and ResNet50	Accuracies of VGG16 – 96, InceptionV3 – 78, ResNet50 – 95 Precision of VGG16 – 94, InceptionV3 – 75, ResNet50 – 92 Recall of VGG16 – 100, InceptionV3 – 70, ResNet50 – 89 F1-score of VGG16 – 98, InceptionV3 – 73, ResNet50 – 94
^34^	Custom MRI dataset collected and augmented by Swati Kanchan from NIT Durgapur	Transfer learning and fine-tuning of MobileNet CNN; image resizing and normalization; GradCam for visual explanation	Fine-tuned MobileNet CNN	Validation Accuracy: 97.24; Test Accuracy: 97.86; Precision: 97.91; Recall: 97.86; F1-score: 97.86 for four class classification
**Our** **Approach**	PPMR dataset^6^	Image processing pipeline using grayscale conversion, Min–Max normalization, histogram equalization, bilateral filtering, and Canny edge Detection	Modified DenseNet-201 and MobileNetV2	Accuracies of DenseNet – 100, MobileNet – 99.8 Precision of DenseNet – 100, MobileNet – 99.6 Recall of DenseNet – 100, MobileNet – 100

## Conclusion

Polymicrogyria (PMG) is a neurological disorder that needs to be detected accurately for the better treatment of children suffering from this disorder. This study elucidates the profound impact of image pre-processing on the classification accuracy of Convolutional Neural Networks (CNNs) in the detection of Polymicrogyria (PMG) from MRI images. The proposed pre-processing pipeline—which combines Bilateral filtering, Min–Max normalization, Contrast Limited Adaptive Histogram Equalization (CLAHE), and Canny edge detection consistently enhanced the performance of various CNN architectures. The key research findings reveal a significant accuracy improvement across multiple models, with ResNet-50, ResNet-101, and VGG-16 exhibiting the most substantial gains of 1.22%, 10.3%, and 3.43% on the test set, respectively.

The pre-processing techniques demonstrated efficacy in unveiling relevant image features while mitigating noise, thereby enabling CNNs to learn more discriminative representations. The results showed a varying degree of improvement across different models, with ResNet and VGG architectures benefiting more substantially compared to MobileNetV2 and DenseNet-201. GradCAM++ analysis indicates that the models focus on anatomically appropriate structures, confirming that the pre-processing approach effectively highlights the subtle structural abnormalities characteristic of PMG. These results emphasize the critical role of tailored image pre-processing in medical image analysis, especially for intricate tasks such as PMG detection in MRI scans. By optimizing the input data, we have demonstrated that even state-of-the-art CNN architectures can achieve notable performance gains. However, given the single-dataset validation and limitations discussed, the proposed methodology shows potential as a decision-support tool for healthcare providers. The methodology can provide visualization enhancement and automated detection assistance to help validate clinicians’ diagnostic findings and potentially identify missed cases, ultimately contributing toward more accurate diagnoses of this complex neurological condition.

## Data Availability

The data that support the findings of this study are available from the corresponding author upon reasonable request.
